# The TEOGIC study project: a comprehensive characterization of early onset gastrointestinal cancer in the Northern area of Spain

**DOI:** 10.1186/s12885-024-12454-9

**Published:** 2024-06-01

**Authors:** R. Vera, N. Castro, I. Labiano, A. Lecumberri, A. E. Huerta, H. Arasanz, I. Caseda, F. Ruiz-Pace, C. Viaplana, V. Arrazubi, I. Hernandez-Garcia, E. Mata, D. Gomez, S. Laguna, J. Suarez, I. Fernandez-De-los-Reyes, M. Rullan, F. Estremera, V. Alonso, R. Pazo-Cid, A. Gil-Negrete, A. Lafuente, A. Martin-Carnicero, R. Dienstmann, M. Alsina

**Affiliations:** 1 Oncobiona Group, Navarrabiomed-Instituto de Investigación Sanitaria de Navarra (IdiSNA), Pamplona, Spain; 2 Department of Medical Oncology, Hospital Universitario de Navarra (HUN), Pamplona, Spain; 3 Oncology Data Science Group, Vall d’Hebron Institute of Oncology (VHIO), Barcelona, Spain; 4 Department of Surgery, Hospital Universitario de Navarra (HUN), Pamplona, Spain; 5 Department of Pathology, Hospital Universitario de Navarra (HUN), Pamplona, Spain; 6 Molecular Pathology of Cancer Group, Navarrabiomed, Universidad Pública de Navarra (UPNA), Instituto de Investigación Sanitaria de Navarra (IdiSNA), Pamplona, Spain; 7 Department of Gastroenterology and Hepatology, Hospital Universitario de Navarra (HUN), Pamplona, Spain; 8 Digestive System and Metabolism Diseases Group, Instituto de Investigación Sanitaria de Navarra (IdiSNA), Pamplona, Spain; 9 Department of Medical Oncology, Hospital Universitario Miguel Servet, IISA, Zaragoza, Spain; 10 Department of Medical Oncology, Hospital Universitario Donostia, San Sebastian, Spain; 11 Department of Medical Oncology, Hospital San Pedro, Logroño, Spain; 12 University of Vic – Central University of Catalonia, Vic, Spain

**Keywords:** Gastrointestinal cancer, Colorectal cancer, Gastroesophageal cancer, Pancreatic cancer, Early onset gastrointestinal cancer, Environmental risk factors, Microbiome, Tumor microenvironment, Precision medicine

## Abstract

**Background:**

Gastrointestinal cancers represent one of the most prevalent diseases worldwide. Strikingly, the incidence of Early Onset Gastrointestinal Cancer (EOGIC) has been rising during the last decades and changes in lifestyle and environmental exposure seem to play a role. EOGIC has been defined as a different entity compared to on-average gastrointestinal cancer, with distinct clinical and molecular characteristics. Inherent to the particularities of younger age, there is an unmet need for a tailored approach for the management of these patients. The TEOGIC proposes a comprehensive study to characterize EOGIC patients in the northern of Spain.

**Methods:**

Patients with histologically confirmed new diagnosis of colorectal, gastroesophageal and pancreatic adenocarcinoma will be considered for two cohorts: EOGIC (≤ 50 years old) and non-EOGIC (60–75 years old), with a ratio of 1:2. Two hundred and forty patients will be recruited in 4 Public Hospitals from northern Spain. After receiving unified informed consent, demographic and clinical data of the patients will be collected in a REDCap database. Lifestyle related data will be obtained in questionnaires assessing diet, physical activity and the general quality of life of the patients before diagnosis. Biological samples prior to any onco-specific treatment will be obtained for the analyses of circulating inflammatory proteins, gut microbiota, and the proteome of the tumor microenvironment. Histologic characteristics and routine biomarkers will be also collected. Thereafter, data will be integrated and analyzed to assess tumor specific, pan-tumor and sex-associated differential characteristics of EOGIC.

**Discussion:**

The underlying risk factors and differential characteristics of EOGIC remain poorly studied, particularly in our geographical area. Although limited by the exploratory nature and the small sample size estimated to be recruited, TEOGIC represents the first attempt to comprehensively characterize these young patients, and thus attend to their special needs. Findings derived from this study could contribute to raise awareness and preventive behaviors in the population. In parallel, molecular studies could lead to the identification of potential novel non-invasive biomarkers and therapeutic targets that would help in the development of the tailored clinical management of these patients, focusing on screening programs for early diagnosis and precision medicine.

## Background

Gastrointestinal (GI) cancer includes malignant tumors of the GI tract such as colon, rectum, esophagus, stomach, pancreas, liver and biliary tract. In 2020, colorectal was the cancer with the third highest incidence and the second cause of cancer-related deaths worldwide. The global incidence and prevalence of other GI cancers was also relevant that year, in this regard, both gastroesophageal and pancreatic cancer represented 11.7% of all new cancer diagnoses and 17.9% of all cancer-related deaths [1]. In Spain, estimated incidences by 2024 ranked colorectal, pancreatic and gastroesophageal as the first, seventh and ninth most incident cancers, respectively [2]. Even though a global decreasing incidence of colorectal and gastroesophageal cancer is observed, some studies show an increase in Early-Onset GastroIntestinal Cancer (EOGIC), defined as that diagnosed in the population below 50 years old. Considering pancreatic cancer, both on average-onset and early-onset incidences are gradually growing [3–6]. Specific data regarding cancer incidence among young adults are still lacking in our country.

Several studies have assessed the occurence of germline mutations in patients with EOGIC [7, 8], however only a small fraction is linked to a hereditary cancer predisposition syndrome, since most of EOGIC cases are sporadic. Therefore, the increase in EOGIC incidence in the last years seems to be influenced by environmental factors such as changes in diet and lifestyle. Obesity, sedentary habits and some dietary patterns are well-known cancer risk factors and are prevalent among young adults developing GI tumors [5, 9–12]. The increased incidence in developed countries of early onset colorectal cancer (EOCRC) or gastroesophageal cancer (EOGEC) also support the hypothesis of environmental factors playing a role in this trend. In this regard, alterations in the gut microbiota due to antibiotic consumption, eradication of *H. pylori* infections or autoimmune atrophic gastritis have been associated to EOCRC and EOGEC [3, 13–16].

Some studies have assessed differential clinical and molecular characteristics of EOGIC, proposing the recognition of a novel entity with special requirements for diagnosis and clinical management. EOGIC usually presents in advanced clinical stages and with aggressive phenotypes, including poor differentiation, signet ring histology and vascular-perineural invasion [17]. EOCRC typically occurs in the distal colon and rectum, with a higher proportion of microsatellite unstable tumors, fewer cases of mutations in *APC*, *KRAS*, and *BRAF*^V600E^, and mostly with a molecular subtype related to infiltration of inmune cells [18–20]. Interestingly, it has been linked to a distinctive microbiota [21]. EOGEC commonly associates with atrophic gastritis from autoinmune phenomenons. It presents higher tumor grades, higher proportion of poorly cohesive histology and signet-ring cell subtype, with an increment of CDH1 mutations, as well as the molecular subtype EBV (TCGA classification) [20, 22]. Early onset pancreatic cancer has lower proportion of *KRAS*, while *SMAD4* mutations are more frequent. Different signaling pathways like Hedgehod, TGFβ, hypoxia or PI3K/AKT have also been reported in these patients [23, 24].

Additionally, EOGIC patients have distinctive characteristics that affect their clinical management. Due to their extended life expectancy, they face long-term effects from therapeutic strategies, which may impact their quality of life and more specifically their sexual and reproductive health. Furthermore, cancer diagnosis at a young age normally represents a more pronounced psychological disorder derived from its impact on professional, social and familiar commitments. This is why it is essential to treat these patients individually, considering all these factors and providing a more personalized and precise approach [6, 25–30].

In summary, available evidence suggests that environmental factors underlie most of the increased incidence of EOGIC, although with geographical differences [3]. Available studies also suggest that EOGIC represents a distinct entity with differential molecular signatures that affect the tumor physiopathology and patients’ outcomes. Besides, young patients need a distinct approach considering their longer life expectancy and unique familiar and social commitments. In Spain, comprehensive studies focused on the incidence and characterization of this population are still lacking. In a retrospective study analyzing locally advanced rectal cancer recently published by our group, we observed that patients diagnosed at a young age (≤ 50 years old) showed higher rates of response to neoadjuvant therapy compared to older patients, which suggests that these patients could benefit from a tailored clinical management [31].

## TEOGIC study methods

### Study design and aim

TEOGIC is an observational, transversal and multicenter study with the objective of characterizing EOGIC (i.e. diagnosed in patients ≤ 50 years old) in comparison to non EOGIC from a comprehensive approach, covering aspects from tumor biology to patients´ lifestyle.

Project investigators comprise a multidisciplinary team involving Public Hospitals, a Biomedical Research Center, and a research group specialized in oncology data science. The project is led by the Health Research Institute of Navarra (IdiSNA), including the University Hospital of Navarra (HUN)-Pamplona-Iruña and the Biomedical Research Center Navarrabiomed. Three more Hospitals of the Northern area of Spain participate in patient recruitment: Miguel Servet Hospital (HMS)-Zaragoza, University Hospital Donostia (HUD)-San Sebastián-Donostia and San Pedro Hospital (HSP) Logroño. ODysSey, from the Vall d´Hebron Institute of Oncology (VHIO)-Barcelona will be in charge of the management, integration and interpretation of multiomics and clinical data.

### Patient selection and recruitment

Patients will be selected and recruited in routine medical appointments by specialized medical oncologists at the participating Hospitals during the period of 01/2024–12/2025. Patients will be included consecutively based on inclusion and exclusion criteria, following a ratio of 1 EOGIC: 2 non-EOGIC.

#### Inclusion criteria

- Patients with newly diagnosed, histologically confirmed colorectal adenocarcinoma (CRC, group A), gastroesophageal adenocarcinoma (GEC, group B) and pancreatic adenocarcinoma (PC, group C).
- Age 18–50 (both included) years old for the EOGIC group, and 60–75 (both included) years old for the non-EOGIC group.

#### Exclusion criteria

- Eastern Cooperative Oncology Group (ECOG) performance status of ≥ 3.
- Presence of comorbidities which may compromise participation of the patient in the study (mainly in terms of capability to complete the questionnaires).
- Having received oncological treatment directed at the newly diagnosed cancer (i.e., at the time of inclusion the patient has not yet started any type of treatment, including surgery).

Although there is a lack of consensus in the age threshold to define EOGIC, it is generally set at 50 years old, in agreement with the initiation of most of the screening programs [6]. Regarding the non-EOGIC group, we set the lower age at 60 years old, in order to ensure a clear differentiation between groups; while the upper limit is established at 75 years old in order to exclude the very elderly population. Again, although the lack of consensus in the age to define elderly patients, several studies in the field consider 75 years old as the lower limit to define this population, and present evidence on their differential characteristics compared to on-average patients [32–36]. Age range is also defined with the objective to minimize age related confounding effect.

**Fig. 1:**
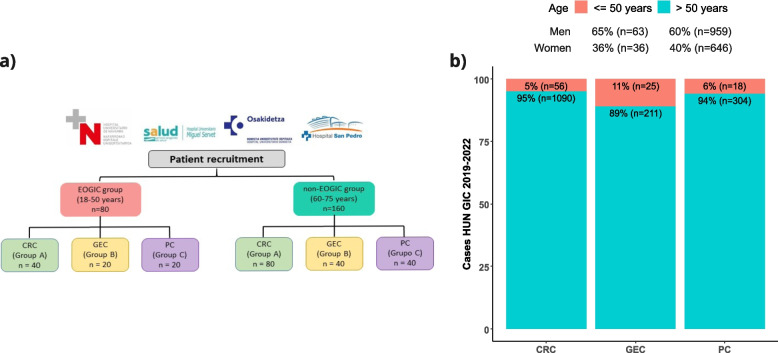
Estimated patient recruitment. **a** Diagram of the recruiting centers and estimated number of patients to be recruited by group. **b** Number of cases with GI cancer treated in Medical Oncology Unit of the HUN in the years 2019–2022. Red depicts EOGIC, while green depicts non-EOGIC (age > 50 years) CRC: colorectal cancer; EOGIC: early onset gastrointestinal cancer. GEC: gastroesophageal cancer; PC: pancreatic cancer

We estimate the following number of patients to be included in each group: CRC-group A, 40 EOGIC and 80 non-EOGIC; GEC- group B, 20 EOGIC, 40 non-EOGIC; PC-group C, 20 EOGIC, 40 non-EOGIC (Fig. 1a). Estimated number of patients has been calculated based on incidence of EOGIC in HUN in the years 2019–2022 (Fig. 1b), and assuming a similar incidence in the other participating hospitals, taking into account that all participating Hospitals cover a similar target population. The rate of patient inclusion will be evaluated periodically and if necessary, recruitment period will be extended or additional Hospitals of the geographical area will be invited to participate in the study in order to increase patient recruitment.

As aforementioned, patients will be included in a ratio of 1 EOGIC: 2 non-EOGIC with the aim of increasing sample-size in the control group, thus optimizing statistical analyses and ensuring study viability. Assuming a statistical power of 80% and a significance level of 5%, this sample size allows for the detection of statistically significant differences between the EOGIC and the non-EOGIC group in an exploratory approach. For continuous variables at a Cohen´s d of 0.385-medium size effect; and for categorical variables, with variables at a 10% prevalence in the non-EOGIC group, a15% of size-difference between groups; with variables at a 30–70% prevalence in the non-EOGIC group, a 20% difference between groups. Additionally, semi-supervised automatic learning will be employed for pan-tumor comparisons, a sample size of 60 EOGIC and 140 non-EOGIC patients is necessary to detect differences in an interest cluster of 20%.

This study is in agreement with international, national and local regulations for clinical studies (latest version of the Declaration of Helsinki, The Spanish Law for Biomedical Research and local regulations “Normas de Buena Práctica Clínica y Orden Foral 125/2009) as well as personal data protection (General Data Protection Regulation (RGPD-Regulation (EU) 2016/679), Spanish Organic Law 3/2018 and the local regulation “Resolution 1387–2017 of the Managing Director of the SNS-O”). Ethics Committee for Investigation with medicinal products from Navarra, have approved this project and a unified informed consent and patient-information sheet has been developed following guidelines of the Spanish Medication and Sanitary Product Agency (AEMPS). Previous to the inclusion, informed consent will be obtained for each patient.

### Data collection and analyses

**Fig. 2:**
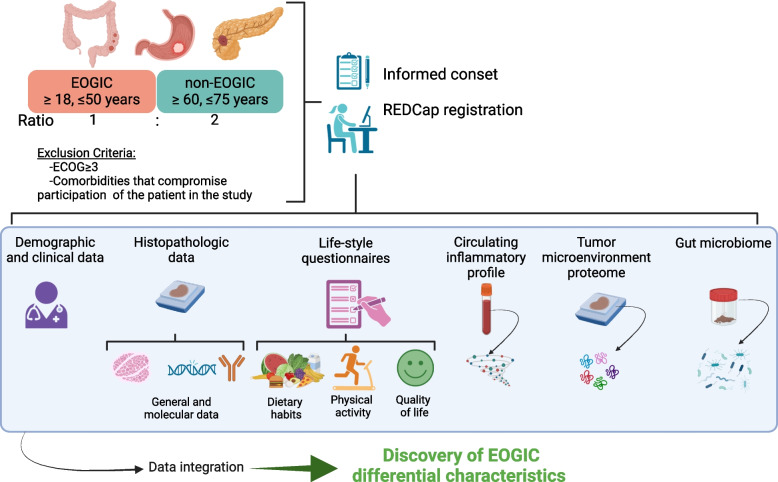
General diagram of the study. Diagram summarizing patient recruitment criteria and data to be recorded in the TEOGIC study. EOGIC: early onset gastrointestinal cancer; ECOG: Eastern Cooperative Oncology Group

Figure 2 shows a general outlook of the study. After signing the informed consent, patients will be registered and study data will be collected and managed using REDCap electronic data capture tools hosted at VHIO internal server [37, 38]. An automated ID will be generated for each patient. Demographic, clinical and histopathological data will be recorded; patient-reported questionnaires will be collected, and biological samples will be obtained for further analyses.

#### Demographic and clinical data and histopathological data

Recruiting medical personnel will be in charge of obtaining the variables in REDCap via a complete clinical interview and screening of the digital clinical record.

Demographic information related to the patients’ environment will be recorded, including, town of residence, country of origin and year of migration if applicable, housing status, social network, educational level and occupation.

In parallel, a comprehensive clinical profile of the patient will also be recorded, including ECOG Performance Status at diagnosis, height, current and usual body weight, body mass index, waist-hip ratio, allergy record, current medication, non-steroidal anti-inflammatory drug (NSAID) and antibiotics consumption, toxic habits, co-morbidities (*H. Pylori* infections and treatment, particularly for patients with GEC), previous surgeries in the affected organs, previously diagnosed malignancies and familial oncologic history as well as gynecological history for women. Additionally, a complete blood analysis covering a hemogram and a complete biochemistry as well as the tumoral markers carcinoembryonic antigen (CEA) and Carbohydrate antigen 19–9 (CA 19–9) will also be included.

#### Histopathological data and tissue proteome

General histopathological and molecular characteristics of the tumor will be comprehensively studied, via specific determinations relevant for each of the etiologies.

General histopathological characteristics, tumor location and clinical tumor stage will be recorded. Colorectal and gastroesophageal cancer staging will be assessed according to The Eighth Edition AJCC Cancer Staging Manual [39]. Pancreatic cancer will be classified as resectable, borderline resectable, locally advanced unresectable and metastasic. Number of metastasic sites and their location will also be recorded in stage IV of the disease. Histology type and differentiation grade will be assessed in formalin-fixed paraffin-embedded (FFPE) tissue. Molecular characteristics to be recorded in FFPE tissue include MMR/MSI status by immunohistochemical analysis of mismatch-repair proteins (*MLH1, MSH2, MSH6*, and *PMS2*) as well as CD3 and CD8 infiltration in all tumors. Additional molecular characteristics will also be measured according to tumor type: *HER2* amplification status, *KRAS*, *NRAS* and *BRAF* mutations for CRC; *HER2* overexpression/amplification status, PDL1 (CPS) and CLDN 18.2 and FGFR2b expression for GEC; and *KRAS* mutations for PC. For all tumors, Next-Generation Sequencing (NGS) analyses targeting cancer-related genes will be performed if a suitable sample is available.

Histopathological characteristics will be preferentially assessed in the recruitment center. If available after routine determinations for diagnosis and clinical management, FFPE tissue samples will be sent to the Pathology Department of HUN in order to perform a centralized assessment of missing determinations.

#### Lifestyle associated variables

Questionnaires related to dietary habits, physical activity and general quality of life will be collected for each patient.

*Dietary habits questionnaire*: a food-frequency questionnaire (FFQ) consisting on 136 items that evaluate intake of total calories, protein, carbohydrates, fats (saturated, mono and polyunsaturated), alcohol, cholesterol, fiber, vitamin A and vitamin C in the last year will be employed. This questionnaire was developed fully in Spanish and based on dietary habits in the country [40], since its development it has also been updated in agreement with food composition tables for Spain [41]. Due to its complexity it requires the assistance of trained personnel for its completion [40]. The questionnaire has been validated in several epidemiological studies, including studies in patients with cancer [41–44]. This questionnaire will always be answered with the assistance of trained personnel via telephone calls with the patient.

*Physical activity questionnaire*: the global physical activity questionnaire (GPAQ) developed by the World Health Organization consisting of 16 items that evaluate physical activity and sedentary behavior will be employed. This questionnaire has been extensively validated, also in patients with cancer [45, 46] and in its Spanish version [47]. Although initially developed for face-to-face interviews with trained personnel, several studies have validated administration of the questionnaire as self-reported [48–51].

*Quality of life questionnaire*: the short form health survey (SF-36) will be employed. The SF-36 questionnaire was developed in the USA to assess general health status in the Medical Outcomes Study [52]. Since then, it has been translated to numerous languages including Spanish [53, 54] and has been extensively validated in patients with cancer [55–57], within the Spanish context [58–60]. This questionnaire was initially developed to be self-reported [52], however, several studies have also reported its validity via telephone-calls or online [61–63].

In this study, the FFQ questionnaire will always be completed with the assistance of trained personnel via previously scheduled telephone calls with the patients. The GPAQ and SF-36 will be self-reported, and when needed, patients will be offered the assistance of trained personnel via telephone calls.

Of note, GPAQ and SF-36 questionnaires are designed to assess physical activity and quality of life at the time of questionnaire completion. Nevertheless, in this study, patients will be recruited after the diagnosis of cancer, which may heavily affect patients´ lifestyle. Moreover, it is the focus of this study to compare EOGIC and non-EGOIC patient´s general lifestyle and its impact on EOGIC development. Therefore, questionnaires will be adapted to evaluate patients´ lifestyle 1 year before diagnosis.

#### Circulating inflammatory protein profile

The circulating inflammatory protein profile will be obtained for each patient. For this purpose, blood samples will be obtained at the time of recruitment, and will always be treatment-naïve including surgery (i.e. samples will obtained before any kind of onco-specific therapeutic intervention, including resection intended surgery). A sample of 10 ml of total blood in vacutainer® EDTA tubes will be drawn by trained nurses. Thereafter, samples will be processed for plasma separation within 2 h since collection, following standardized work protocols in agreement with the Biobank of Navarrabiomed, and stored at -80ºC. Samples will be sent to Navarrabiomed-HUN for centralized storage and subsequent analyses.

In order to assess the circulating inflammatory profile of the patients, a panel of 96 key proteins involved in tumoral and inflammatory processes will be assessed (Olink® Target 96 Immuno-Oncology) by the proximity extension assay (PEA) technology.

#### Feces samples: microbiome analysis

Fecal microbiome analyses will be conducted by the sequencing the *16S* gene. Feces samples will be obtained at the time of recruitment, and will always be treatment-naïve including surgery. Samples will be self-collected by the patients following detailed instructions with the DANASTOOL Sample Collection MICROBIOME kit (Danagen). This kit consists of a sample collection instrument and a tube holding a stabilization solution that enables stabilization of DNA for several months at room temperature and indefinitely at -80ºC. Upon reception, feces samples will be homogenized and stored at -80ºC, thereafter they will be sent to Navarrabiomed-HUN for further processing and analyses.

DNA extraction will be conducted using the DANASTOOL Microbiome Fecal DNA kit (Danagen), and the quantity and quality of the extracted DNA will be assessed with NanoDrop ND-1000 (Thermo Fisher Scientific) and the Qubit Fluorometer (Thermo Fisher Scientific), and stored at -80ºC. For microbiome analyses, libraries will be prepared with the xGen Amplicon Panels for 16S and IST1 kit (IDT), which allows for the PCR-based enrichment on the *16S* v2 V1-V9 rRNA regions. Thereafter, sequencing will be carried out on the Illumina NovaSeqplatform. After quality control (QC) assessment, data will be processed with QIIME to obtain Operational Taxonomic Units (OTUs) against reference databases (Greengenes or Silva).

#### Tumor microenvironment proteomic analysis

FFPE tissue samples will be obtained from diagnostic biopsies or resection specimens naive of treatment. Proteomic analyses will be performed after all needed determinations for diagnosis and clinical management have been conducted. Proteomic analyses will be conducted in the Proteomics unit of Navarrabiomed (ISO9001:2015 certified). Samples will undergo "in-gel" digestion, peptide reconstitution, and purification before identification by liquid chromatography-tandem mass spectrometry (LC–MS/MS), using the Ultimate 3000 RSLCnano system coupled with an Orbitrap Exploris 480 mass spectrometer (Thermo Fisher Scientific). Spectra resulting from the analysis will be processed using MaxQuant software and compared with the most updated *H.sapiens* reference proteome from the UniProt database.

#### Statistical analyses

Descriptive and comparative analyses will be performed separately for each type of tumor: CRC (Group A), GEC (Group B), and PC (Group C) as well as at a pan-tumor level, comparing all EOGIC to non-EOGIC patients.

Population characteristics will be described using measures of central tendency and dispersion for continuous variables and frequencies and percentages for categorical variables. Normality of the sample will be analyzed for continuous variables through histogram exploration, the Shapiro–Wilk test, and homoscedasticity by Levene's test. Group comparisons will be made using Student's t-test or Mann–Whitney U test for continuous variables and Pearson's Chi-square test or Fisher's exact test for categorical variables. Correlation between different measurements and conditions of interest will be assessed using Pearson's or Spearman's correlation tests.

Analyses for circulating inflammatory proteins, the microbiota and the tumor microenvironment proteome, will be conducted utilizing specialized software for each application. Raw data will be processed, applying quality criteria, and differential signatures between groups will be identified. For signature identification, previously mentioned tests, including correction methods for multiple comparisons such as the false discovery rate (FDR).

For pan-tumor and gender/sex-level comparisons, machine learning algorithms will be used, allowing integration of all patient clinical and lifestyle profile data with variables derived from circulating inflammatory proteins, microbiome and tumor microenvironment proteome profiles, identifying clusters of patients with similar patterns.

Statistical analysis will be performed using IBM Statistical Package for the Social Sciences (SPSS) version 24.0, GraphPad Prism v9, and R 4.1.0 (R Foundation for Statistical Computing, Vienna, Austria. http://www.R-project.org/).

## Discussion

EOGIC incidence is rising worldwide at an alarming rate and due to the relatively low proportion of hereditary cases, it is thought to be associated with changes in lifestyle and environmental exposure [6]. Some studies have shown that EOGIC has differential clinical and molecular features [6, 8, 18, 20, 24], but prospective and comprehensive studies are still lacking, especially in our geographical area [3, 4].

Patients with EOGIC represent an especially vulnerable population, with distinct challenges and needs. The diagnosis of EOGIC can lead to the interruption of education, professional activity and/or familial responsibilities and have a significant effect on their overall quality of life. Cancer associated morbidity is also distinctive in these patients that may have to face premature mortality. Moreover, the longer life expectancy of these patients requires special attention to the long-term side effects of chemo-radiotherapy treatments, especially those affecting cardiovascular, reproductive and sexual capacity; as well as the effect on body image after aggressive surgeries [6, 25–30].

The TEOGIC study aims to characterize the distinctive features of EOGIC through a comprehensive approach that considers parameters encompassing clinical and demographic, as well as lifestyle characteristics of the patient, together with the biological characteristics of the tumor. The identification of unique biomarkers holds the potential for integration across preventive and therapeutic levels, contributing to the development of precision medicine. Our overarching goal is to enhance the prognosis and quality of life for EOGIC patients. This study has the potential to significantly influence the prevention and awareness of gastrointestinal cancer development within the population. By identifying lifestyle-associated factors linked to EOGIC, we can enhance preventive measures. Additionally, our molecular studies (microbiome and circulating proteins) could contribute to the identification of new sources of non-invasive biomarkers with a potential for early diagnosis and screening programs currently lacking in gastroesophageal and pancreatic cancer [17]. Similarly, the characterization of the proteome of the tumor microenvironment could unravel novel potential therapeutic targets and thus lead to the generation of new hypotheses in need of validation in upcoming studies.

The main limitations of the present study are the inclusion of a relatively small number of patients and a recruitment restricted to a limited geographical area. The TEOGIC study represents the first initiative to characterize EOGIC in our area and would serve as the founding experience to establish the collaborations and synergies of multidisciplinary teams that would enable the development of extended studies in the future. Results derived from this study will also contribute to raising awareness about the importance of new risk factors in the development of EOGIC and thus, serve to promote lifestyle changes in the general population. Additionally, as aforementioned, the TEOGIC project features an exploratory nature to finding new sources of biomarkers and therapeutic targets.

## Conclusion

TEOGIC is a transversal study of Early-Onset Gastrointestinal Cancer conducted across four hospitals within the national public healthcare system, a biomedical research center, and a research group specialized in data integration and interpretation in oncology. Its primary objective is to identify distinctive characteristics associated with patients with EOGIC. The findings from this study are expected to have an impact in preventive measures and therapeutic interventions in this group of patients.

## Data Availability

No datasets were generated or analysed during the current study.

## Abbreviations

*APC*: Adenomatous polyposis coli
CA19-9: Carbohydrate antigen 19–9
CEA: Carcinoembryonic antigen
CLDN 18.2: Claudin 18.2
CRC: Colorectal cancer
CDH1: Cadherin 1
EBV: Epstein-Barr virus
ECOG: Eastern Cooperative Oncology Group
EOGIC: Early onset Gastrointestinal Cancer
EOCRC: Early onset colorectal cancer
EOGEC: Early onset gastroesophageal cancer
FDR: False discovery rate
FFQ: Food frequency questionnaire
FFPE: Formalin-fixed paraffin-embedded
FGFR2b: Fibroblast growth factor receptor 2b
GPAQ: Global physical activity questionnaire
GEC: Gastroesophageal cancer
GI: Gastrointestinal cancer
HMS: Miguel Servet Hospital
HSP: San Pedro Hospital
HUD: University Hospital Donostia
HUN: University Hospital of Navarra
*KRAS*: Kirsten rat sarcoma virus
MMR: Mismatch repair
MSI: Microsatellite instability
NSAID: Non-steroidal anti-inflammatory drug
OTUs: Operational Taxonomic Units
PC: Pancreatic cancer
PDL1: Programmed death-ligand 1
PEA: Proximity extension assay
PIK3: Phosphatidylinositol 3-kinase
QC: Quality control
TEOGIC: Transversal study on early-Onset Gastrointestinal Cancer
TGFβ: Transforming growth factor β
USA: United stated of America
VHIO: Vall d´Hebron Institute of Oncology

## References

[1] Sung H, Ferlay J, Siegel RL, Laversanne M, Soerjomataram I, Jemal A (2021). Global Cancer Statistics 2020: GLOBOCAN estimates of incidence and mortality worldwide for 36 cancers in 185 countries. CA Cancer J Clin.

[2] (SEOM) SEdOM. Las Cifras del Cancer en España 2024. https://seom.org/ejercicio-contra-el-cancer/las-cifras-del-cancer-en-espana-20242024. Available from: https://www.seom.org/images/LAS_CIFRAS_2024.pdf.

[3] Siegel RL, Torre LA, Soerjomataram I, Hayes RB, Bray F, Weber TK (2019). Global patterns and trends in colorectal cancer incidence in young adults. Gut.

[4] Araghi M, Soerjomataram I, Bardot A, Ferlay J, Cabasag CJ, Morrison DS (2019). Changes in colorectal cancer incidence in seven high-income countries: a population-based study. Lancet Gastroenterol Hepatol.

[5] Sung H, Siegel RL, Rosenberg PS, Jemal A (2019). Emerging cancer trends among young adults in the USA: analysis of a population-based cancer registry. Lancet Public Health.

[6] Ben-Aharon I, van Laarhoven HWM, Fontana E, Obermannova R, Nilsson M, Lordick F (2023). Early-onset cancer in the gastrointestinal tract is on the rise-evidence and implications. Cancer Discov.

[7] Liao H, Cai S, Bai Y, Zhang B, Sheng Y, Tong S (2021). Prevalence and spectrum of germline cancer susceptibility gene variants and somatic second hits in colorectal cancer. Am J Cancer Res.

[8] Pocurull A, Herrera-Pariente C, Carballal S, Llach J, Sánchez A, Carot L (2021). Clinical, molecular and genetic characteristics of early onset gastric cancer: analysis of a large multicenter study. Cancers (Basel)..

[9] Lauby-Secretan B, Scoccianti C, Loomis D, Grosse Y, Bianchini F, Straif K (2016). Body fatness and cancer-viewpoint of the IARC Working Group. N Engl J Med.

[10] Nguyen LH, Liu PH, Zheng X, Keum N, Zong X, Li X (2018). Sedentary Behaviors, TV Viewing Time, and Risk of Young-Onset Colorectal Cancer. JNCI Cancer Spectr..

[11] Nguyen LH, Cao Y, Hur J, Mehta RS, Sikavi DR, Wang Y (2021). The sulfur microbial diet is associated with increased risk of early-onset colorectal cancer precursors. Gastroenterology.

[12] Carroll KL, Frugé AD, Heslin MJ, Lipke EA, Greene MW (2022). Diet as a risk factor for early-onset colorectal adenoma and carcinoma: a systematic review. Front Nutr.

[13] Petrillo A, Federico P, Marte G, Liguori C, Seeber A, Ottaviano M (2023). Non-hereditary early onset gastric cancer: an unmet medical need. Curr Opin Pharmacol.

[14] Song H, Held M, Sandin S, Rautelin H, Eliasson M, Söderberg S (2015). Increase in the prevalence of atrophic gastritis among adults age 35 to 44 years old in Northern Sweden between 1990 and 2009. Clin Gastroenterol Hepatol.

[15] Arnold M, Ferlay J, van Berge Henegouwen MI, Soerjomataram I (2020). Global burden of oesophageal and gastric cancer by histology and subsite in 2018. Gut.

[16] Chen C, Yang Y, Li P, Hu H (2023). Incidence of gastric neoplasms arising from autoimmune metaplastic atrophic gastritis: a systematic review and case reports. J Clin Med..

[17] Fontana E, Meyers J, Sobrero A, Iveson T, Shields AF, Taieb J (2021). Early-onset colorectal adenocarcinoma in the IDEA database: treatment adherence, toxicities, and outcomes with 3 and 6 months of adjuvant fluoropyrimidine and oxaliplatin. J Clin Oncol.

[18] Zaborowski AM, Abdile A, Adamina M, Aigner F, d'Allens L, Allmer C (2021). Characteristics of early-onset vs late-onset colorectal cancer: a review. JAMA Surg.

[19] Lieu CH, Golemis EA, Serebriiskii IG, Newberg J, Hemmerich A, Connelly C (2019). Comprehensive genomic landscapes in early and later onset colorectal cancer. Clin Cancer Res.

[20] Willauer AN, Liu Y, Pereira AAL, Lam M, Morris JS, Raghav KPS (2019). Clinical and molecular characterization of early-onset colorectal cancer. Cancer.

[21] Kong C, Liang L, Liu G, Du L, Yang Y, Liu J (2023). Integrated metagenomic and metabolomic analysis reveals distinct gut-microbiome-derived phenotypes in early-onset colorectal cancer. Gut.

[22] Pérez-Wert P, Custodio A, Jimenez-Fonseca P, Carmona-Bayonas A, Lecumberri A, Cacho Lavin D (2024). Efficacy and safety of chemotherapy in young patients with advanced gastroesophageal adenocarcinoma: data from the Spanish AGAMENON-SEOM registry. Gastric Cancer.

[23] Varghese AM, Singh I, Singh R, Kunte S, Chou JF, Capanu M (2021). Early-onset pancreas cancer: clinical descriptors, genomics, and outcomes. J Natl Cancer Inst.

[24] Castet F, Fabregat-Franco C, Castillo G, Navarro V, Sierra A, Acosta DA (2023). Clinical and genomic characterisation of early-onset pancreatic cancer. Eur J Cancer.

[25] Incrocci L, Jensen PT (2013). Pelvic radiotherapy and sexual function in men and women. J Sex Med.

[26] Cercek A, Siegel CL, Capanu M, Reidy-Lagunes D, Saltz LB (2013). Incidence of chemotherapy-induced amenorrhea in premenopausal women treated with adjuvant FOLFOX for colorectal cancer. Clin Colorectal Cancer.

[27] Perl G, Nordheimer S, Lando S, Benedict C, Brenner B, Perry S (2016). Young patients and gastrointestinal (GI) tract malignancies - are we addressing the unmet needs?. BMC Cancer.

[28] Zebrack BJ, Mills J, Weitzman TS (2007). Health and supportive care needs of young adult cancer patients and survivors. J Cancer Surviv.

[29] Smith AW, Parsons HM, Kent EE, Bellizzi K, Zebrack BJ, Keel G (2013). Unmet support service needs and health-related quality of life among adolescents and young adults with cancer: the AYA HOPE Study. Front Oncol.

[30] Sanford SD, Zhao F, Salsman JM, Chang VT, Wagner LI, Fisch MJ (2014). Symptom burden among young adults with breast or colorectal cancer. Cancer.

[31] Suarez J, Alsina M, Castro N, Marin G, Llanos C, Oronoz B (2024). Higher rate of pathologic complete response in patients with early-onset locally advanced rectal cancer. ESMO Gastrointestinal Oncol.

[32] Schendel J, Jost E, Mah M, Mack L, McCall M, Gu N (2021). Gastric cancer management in elderly patients: a population-based study of treatment patterns and outcomes in gastric cancer patients ≥ 75 years from Alberta, Canada. Am J Surg.

[33] Natori A, Chan BA, Sim HW, Ma L, Yokom DW, Chen E (2018). Outcomes by treatment modality in elderly patients with localized gastric and esophageal cancer. Curr Oncol.

[34] Hagerty BL, Aversa JG, Dominguez DA, Davis JL, Hernandez JM, McCormick JT (2022). Age determines adjuvant chemotherapy use in resected stage II colon cancer. Dis Colon Rectum.

[35] Henry AC, Schouten TJ, Daamen LA, Walma MS, Noordzij P, Cirkel GA (2022). Short- and long-term outcomes of pancreatic cancer resection in elderly patients: a nationwide analysis. Ann Surg Oncol.

[36] Oba A, Wu YHA, Lieu CH, Meguid C, Colborn KL, Beaty L (2021). Outcome of neoadjuvant treatment for pancreatic cancer in elderly patients: comparative, observational cohort study. Br J Surg.

[37] Harris PA, Taylor R, Thielke R, Payne J, Gonzalez N, Conde JG (2009). Research electronic data capture (REDCap)–a metadata-driven methodology and workflow process for providing translational research informatics support. J Biomed Inform.

[38] Harris PA, Taylor R, Minor BL, Elliott V, Fernandez M, O'Neal L (2019). The REDCap consortium: Building an international community of software platform partners. J Biomed Inform.

[39] Amin MB, Greene FL, Edge SB, Compton CC, Gershenwald JE, Brookland RK (2017). The Eighth Edition AJCC Cancer Staging Manual: continuing to build a bridge from a population-based to a more "personalized" approach to cancer staging. CA Cancer J Clin..

[40] Martin-Moreno JM, Boyle P, Gorgojo L, Maisonneuve P, Fernandez-Rodriguez JC, Salvini S (1993). Development and validation of a food frequency questionnaire in Spain. Int J Epidemiol.

[41] Romanos-Nanclares A, Gea A, Martínez-González M, Zazpe I, Gardeazabal I, Fernandez-Lazaro CI (2021). Carbohydrate quality index and breast cancer risk in a Mediterranean cohort: the SUN project. Clin Nutr.

[42] Gardeazabal I, Ruiz-Canela M, Sánchez-Bayona R, Romanos-Nanclares A, Aramendía-Beitia JM, Shivappa N (2019). Dietary inflammatory index and incidence of breast cancer in the SUN project. Clin Nutr.

[43] Fernandez-Lazaro CI, Martínez-González M, Aguilera-Buenosvinos I, Gea A, Ruiz-Canela M, Romanos-Nanclares A (2021). Dietary antioxidant vitamins and minerals and breast cancer risk: prospective results from the SUN Cohort. Antioxidants (Basel)..

[44] Fernández-Ballart JD, Piñol JL, Zazpe I, Corella D, Carrasco P, Toledo E (2010). Relative validity of a semi-quantitative food-frequency questionnaire in an elderly Mediterranean population of Spain. Br J Nutr.

[45] Baker JL, Di Meglio A, Gbenou AS, El Mouhebb M, Iyengar NM, Michiels S (2022). Association between physical activity and neoadjuvant chemotherapy completion and pathologic complete response in primary breast cancer: the CANTO study. Br J Cancer.

[46] Lee J, Min J, Lee DH, Kang DW, Jeon JY (2021). Intensity- and domain-specific physical activity levels between cancer survivors and non-cancer diagnosis individuals: a propensity score matching analysis. Support Care Cancer.

[47] Ruiz-Casado A, Alejo LB, Santos-Lozano A, Soria A, Ortega MJ, Pagola I (2016). Validity of the physical activity questionnaires IPAQ-SF and GPAQ for cancer survivors: insights from a Spanish Cohort. Int J Sports Med.

[48] Wanner M, Hartmann C, Pestoni G, Martin BW, Siegrist M, Martin-Diener E (2017). Validation of the Global Physical Activity Questionnaire for self-administration in a European context. BMJ Open Sport Exerc Med.

[49] Hoos T, Espinoza N, Marshall S, Arredondo EM (2012). Validity of the Global Physical Activity Questionnaire (GPAQ) in adult Latinas. J Phys Act Health.

[50] Sitthipornvorakul E, Janwantanakul P, van der Beek AJ (2014). Correlation between pedometer and the Global Physical Activity Questionnaire on physical activity measurement in office workers. BMC Res Notes.

[51] Chu AH, Ng SH, Koh D, Müller-Riemenschneider F (2015). Reliability and Validity of the Self- and Interviewer-Administered Versions of the Global Physical Activity Questionnaire (GPAQ). PLoS ONE.

[52] Ware JE, Sherbourne CD (1992). The MOS 36-item short-form health survey (SF-36). I. Conceptual framework and item selection. Med Care..

[53] Alonso J, Prieto L, Antó JM (1995). The Spanish version of the SF-36 Health Survey (the SF-36 health questionnaire): an instrument for measuring clinical results. Med Clin (Barc).

[54] Bullinger M, Alonso J, Apolone G, Leplège A, Sullivan M, Wood-Dauphinee S (1998). Translating health status questionnaires and evaluating their quality: the IQOLA Project approach. International Quality of Life Assessment. J Clin Epidemiol..

[55] Schurr T, Loth F, Lidington E, Piccinin C, Arraras JI, Groenvold M (2023). Patient-reported outcome measures for physical function in cancer patients: content comparison of the EORTC CAT Core, EORTC QLQ-C30, SF-36, FACT-G, and PROMIS measures using the International Classification of Functioning, Disability and Health. BMC Med Res Methodol.

[56] Brown JC, Damjanov N, Courneya KS, Troxel AB, Zemel BS, Rickels MR (2018). A randomized dose-response trial of aerobic exercise and health-related quality of life in colon cancer survivors. Psychooncology.

[57] Hara T, Kogure E, Iijima S, Fukawa Y, Kubo A, Kakuda W (2022). Minimal clinically important difference in postoperative recovery among patients with gastrointestinal cancer. Support Care Cancer.

[58] Vilagut G, Ferrer M, Rajmil L, Rebollo P, Permanyer-Miralda G, Quintana JM (2005). The Spanish version of the Short Form 36 Health Survey: a decade of experience and new developments. Gac Sanit.

[59] Vilagut G, Valderas JM, Ferrer M, Garin O, López-García E, Alonso J (2008). Interpretation of SF-36 and SF-12 questionnaires in Spain: physical and mental components. Med Clin (Barc).

[60] Santillán Coello JM, Sánchez Barrueco Á, González Galán F, Díaz Tapia G, Mahillo Fernández I, Villacampa Aubá JM (2023). Validation of a Spanish chronic obstructive sialadenitis quality of life questionnaire (CSOC). Acta Otorrinolaringol Esp (Engl Ed).

[61] Açma A, Carrat F, Hejblum G (2022). Comparing SF-36 scores collected through web-based questionnaire self-completions and telephone interviews: an ancillary study of the SENTIPAT multicenter randomized controlled trial. J Med Internet Res.

[62] Wettergren L, Mattsson E, von Essen L (2011). Mode of administration only has a small effect on data quality and self-reported health status and emotional distress among Swedish adolescents and young adults. J Clin Nurs.

[63] Basnov M, Kongsved SM, Bech P, Hjollund NH (2009). Reliability of short form-36 in an Internet- and a pen-and-paper version. Inform Health Soc Care.

