# Correction: the TEOGIC study project: a comprehensive characterization of early onset gastrointestinal cancer in the Northern area of Spain

**DOI:** 10.1186/s12885-024-12581-3

**Published:** 2024-07-09

**Authors:** R. Vera, N. Castro, I. Labiano, A. Lecumberri, A. E. Huerta, H. Arasanz, I. Caseda, F. Ruiz-Pace, C. Viaplana, V. Arrazubi, I. Hernandez-Garcia, E. Mata, D. Gomez, S. Laguna, J. Suarez, I. Fernandez-De-los-Reyes, M. Rullan, F. Estremera, V. Alonso, R. Pazo-Cid, A. Gil-Negrete, A. Lafuente, A. Martin-Carnicero, R. Dienstmann, M. Alsina

**Affiliations:** 1 Oncobiona Group, Navarrabiomed-Instituto de Investigación Sanitaria de Navarra (IdiSNA), Pamplona, Spain; 2 Department of Medical Oncology, Hospital Universitario de Navarra (HUN), Pamplona, Spain; 3 Oncology Data Science Group, Vall d’Hebron Institute of Oncology (VHIO), Barcelona, Spain; 4 Department of Surgery, Hospital Universitario de Navarra (HUN), Pamplona, Spain; 5 Department of Pathology, Hospital Universitario de Navarra (HUN), Pamplona, Spain; 6 Molecular Pathology of Cancer Group, Universidad Pública de Navarra (UPNA), Instituto de Investigación Sanitaria de Navarra (IdiSNA), Pamplona, Spain; 7 Department of Gastroenterology and Hepatology, Hospital Universitario de Navarra (HUN), Pamplona, Spain; 8 Digestive System and Metabolism Diseases Group, Instituto de Investigación Sanitaria de Navarra (IdiSNA), Pamplona, Spain; 9 Department of Medical Oncology, Hospital Universitario Miguel Servet, IISA, Zaragoza, Spain; 10 Department of Medical Oncology, Hospital Universitario Donostia, San Sebastian, Spain; 11 Department of Medical Oncology, Hospital San Pedro, Logroño, Spain; 12 University of Vic – Central University of Catalonia, Vic, Spain

**Correction to: Vera et al. BMC Cancer (2024) 24:668** 10.1186/s12885-024-12454-9

Following the publication of the Original Article, the authors identified an error in the surname of one of the co-authors.

**Incorrect**

R. Dientsmann

**Correct**

R. Dienstmann

The author group has been updated above and the Original Article has been corrected.

